# Determinants of maternal health services utilization in urban settings of the Democratic Republic of Congo – A Case study of Lubumbashi City

**DOI:** 10.1186/1471-2393-12-66

**Published:** 2012-07-10

**Authors:** Abel Ntambue ML, Françoise Malonga K, Michèle Dramaix-Wilmet, Philippe Donnen

**Affiliations:** 1 École de Santé Publique, Université de Lubumbashi (ESP/UNILU), Lubumbashi, DRC, Democratic Republic of Congo; 2 École de Santé Publique, département de Biostatistique, Université Libre de Bruxelles, Brussels, Belgium; 3 Centre Scientifique et médical de l’Université Libre de Bruxelles pour ses activités de coopération (CEMUBAC), Brussels, Belgium; 4 École de Santé Publique, département d’Épidémiologie et de Médecine Préventive, Université Libre de Bruxelles, Brussels, Belgium

## Abstract

**Background:**

The use of maternal health services, known as an indirect indicator of perinatal death, is still unknown in Lubumbashi. The present study was therefore undertaken in order to determine the factors that influence the use of mother and child healthcare services in Lubumbashi, Democratic Republic of the Congo.

**Methods:**

This was transversal study of women residing in Lubumbashi who had delivered between January and December 2009. In total, 1762 women were sampled from households using indicator cluster surveys in all health zones. Antenatal consultations (ANC), delivery assisted by qualified healthcare personnel (and delivery in a healthcare facility) as well as postnatal consultations (PNC) were dependent variables of study. The factors determining non-use of maternal healthcare services were researched via logistic regression with a 5% materiality threshold.

**Results:**

The use of maternal healthcare services was variable; 92.6% of women had attended ANC at least once, 93.8% of women had delivered at a healthcare facility, 97.2% had delivered in the presence of qualified healthcare personnel, while the rate of caesarean section was 4.5%. Only 34.6% postnatal women had attended PNC by 42 days after delivery. During these ANC visits, only 60.6% received at least one dose of vaccine, while 38.1% received Mebendazole, 35.6% iron, 32.7% at least one dose of SulfadoxinePyrimethamine, 29.2% folic acid, 15.5% screening for HIV and 12.8% an insecticide treated net.

In comparison to women that had had two or three deliveries before, primiparous and grand multiparous women were twice as likely not to use ANC during their pregnancy. Women who had unplanned pregnancies were also more likely not to use ANC or PNC than those who had planned pregnancies alone or with their partner. The women who had not used ANC were also more likely not to use PNC. The women who had had a trouble-free delivery were more likely not to use PNC than those who had complications when delivering.

**Conclusion:**

In Lubumbashi, a significant proportion of women continue not to make use of healthcare services during pregnancy, as well as during and after childbirth. Women giving birth for the first time, those who have already given birth many times, and women with an unwanted pregnancy, made less use of ANC. Moreover, women who had not gone for ANC rarely came back for postnatal consultations, even if they had given birth at a healthcare facility. Similarly, those who gave birth without complications, less frequently made use of postnatal consultations. As with ANCs, women with unwanted pregnancies rarely went for postnatal visits.

In addition to measures aimed at reinforcing women’s autonomy, efforts are also needed to reinforce and improve the information given to women of childbearing age, as well as communication between the healthcare system and the community, and participation from the community, since this will contribute to raising awareness of safe motherhood and the use of such services, including family planning.

## Background

Antenatal consultations (ANC), skilled attendance at birth, and postnatal care (PNC), which form the objective of this study on maternal and neonatal healthcare services, are among the pillars of safer motherhood. Their efficacy is proven in the prevention of obstetrical risks [1,2] but their use is still lacking. Large gaps still exist between the rich and poor countries on the utilization of these services where, for example, 80% of pregnant women attended ANC at least once in 2007. Less than half followed the recommended number of consultations [3]. Gaps also exist in the provision of antenatal healthcare in the country [4].

In the Democratic Republic of Congo (DRC), 85% of pregnant women attended ANC at least once in 2007. However, this coverage was different between urban areas and rural areas; 92.0% and 80.9% respectively, and only 78.0% in the province of Katanga, which is among the lowest in the country [5].

Whereas the proportion of assisted childbirth is increasing significantly worldwide [3], there is still room for improvement in low-income countries [5]. For example, while in 2007, 80% of deliveries throughout the world took place in the presence of qualified healthcare personnel; this coverage only reached 60% in poor countries [3]. In DRC, this was only 74% and in the province of Katanga 70%; with wide gaps between urban and rural environments. [5].

The reduction of infant mortality in the under-fives is in part linked to that of perinatal mortality. Therefore, the former cannot be expected to reduce without improving the women’s access to perinatal health services [1,6].

Just to take an example of neonatal mortality, Darmstadt et al. [1] showed that when community-based antenatal healthcare at health facilities is used in ideal conditions and in a population with a ANC coverage of 99%, and when this healthcare is coupled with skilled assistance at delivery and postnatal care covering 90% of the population, it is possible to reduce neonatal mortality by over 70%. They also show that this perinatal healthcare is more beneficial and profitable when provided as a package rather than in the form of isolated interventions.

In Lubumbashi, the use of such services, recognised to be an indirect indicator of the perinatal mortality rate, however, remains unknown, which makes it difficult to assess the progress made in pursuit of MDG4-5. Moreover, since the use rate of these services is unknown, the factors explaining this rate are not known either. It is indeed necessary to determine the use rate of mother and child healthcare services in this environment, so that their impact on the maternal-foetal and neonatal prognosis can eventually be evaluated.

The present study was therefore undertaken in order to determine the factors that influence the use of mother and child healthcare services in Lubumbashi, Democratic Republic of the Congo.

## Material and methods

### Context

The study was carried out in the city of Lubumbashi, the Democratic Republic of Congo. Lubumbashi is the administrative centre of Katanga province and the second most populated city in DRC after Kinshasa. It is located in the South East of the DRC, 135 km from the northern boarder of Zambia. Although no census has taken place here since 1984, fore casts within the vaccination programme of 2009 estimated this population to be 1,415,835[7], and the rate of natural growth is as for the whole country at 4% [8]. The city consists of seven administrative districts and eleven healthcare zones; some of which are urban–rural. In order of magnitude, the population is spread as follows in the healthcare zones: Kampemba (276,112), Katuba (200,829), Ruashi (194,355), Lubumbashi (127,071), Kenya (116,766), Kisanga (116,297), Tshiamilemba (111,074), Mumbunda (108,365), Vangu (91,986), Kamalondo (49,393), Kowe (23,587) [7]. Data were collected in specific areas in each of these healthcare zones.

### Study design, sample size and sampling

This was a transversal study carried out in order to determine the use of maternal healthcare services in Lubumbashi. Data were collected between January and February 2010 with help of a questionnaire written in French (interpreted in accordance with the understanding of the interviewee) consisting of closed and semi-open questions administered by surveyors. A pre-survey was carried out in order to test the questionnaire.

The study was carried out on women residing in Lubumbashi who had delivered during the 12 months prior to the survey (January to December 2009). Women who delivered in Lubumbashi during the same period but had not lived there during their pregnancy were excluded from our sample [9]. Women’s informed consent was obtained prior to commencing the interview.

This study was conducted in collaboration with the provincial office of the national program of the reproductive health (PNSR), and it was approved and authorized by the medical ethics committee of the University of Lubumbashi.

Sample size was calculated according to antenatal consultation coverage of 78% in the province of Katanga [5]. With an accuracy of 3% and a confidence level of 95%, a proportion of non-respondents of 10% and a design effect of two, at least 1460 women were recruited for our study [9].

Women were selected by cluster sampling. The clusters were made up of the different healthcare areas. There were 30 clusters for the whole city (Table 1) and clusters were selected by systematic random sampling according to the cumulative frequency of the population that used these services during 2009 [9].

**Table 1:** Number of clusters and subjects surveyed according to healthcare zones

**Healthcare Zone**	**Number of clusters**	**Number of subjects for all of the clusters of the healthcare zone**
Kamalondo	1	50
Kampemba	6	320
Katuba	5	210
Kenya	3	74
Kisanga	3	74
Lubumbashi	3	58
Mumbunda	2	145
Ruashi	5	385
Tshiamilemba	2	145
Total	30	1460

In the same cluster, data collection was carried out systematically according to households using a set inclusion criteria until the limit number required for the healthcare area was reached [9].

Women’s and household data were collected including socio-demographic, attendance at ANC (reasons for attendance and non-attendance, the health interventions received, perceptions of the quality of care received), the place of delivery and the presence of qualified healthcare personnel at the time of delivery, as well as the use of PNC.

Women were interviewed within the households. In order to verify the exactness of the information provided by the women, each one was asked to give the name of the facility where she went for antenatal consultations (ANC). At the facility, the data of the interviewed women were looked up in the registers and records of ANC, and compared with the women’s statements. This procedure was followed as women in Lubumbashi do not have ANC cards on which the frequency and care administered during ANC can be indicated. All such information is usually written in the register and record, which is often kept at the ANC facility. In rare cases where women take the ANC sheet with them, they either tend to forget it at home at the time of childbirth, or they leave it at the maternity home after the birth.

Information regarding the delivery itself was obtained by going to the maternity home. Besides the information provided by each woman, the healthcare facility in which she gave birth was visited to confirm the status of the healthcare staff who attended the birth. At the same time, information regarding complications and the outcome of the delivery was obtained.

For women who gave birth at home, the name of the healthcare practitioner who attended the birth was searched in order to confirm their training and aptitude to attend during childbirth. This information was obtained by interviewing the person in question. The status of personnel qualified to attend childbirth was recognised if, in addition to their basic training as healthcare practitioner, they had attended at least one birth in the month preceding the delivery in relation to which the information is sought. If the person could not be found, the healthcare area or the official nurse of the healthcare centre was contacted to confirm the status of the person who attended the birth.

### Selection of women

In total, 2177 of the households visited had at least one woman who had carried a pregnancy in 2009. Among these, 0.9% (20 women) had miscarried, 0.2% (four) had died during pregnancy, 17.5% (381) were still pregnant at the time of the survey and 81.3% (1772) had already delivered. Among the women who had delivered, 0.2% (three) had died during delivery, 0.2% (four) had died within the 42 days after delivery and 0.2% (three) of the survivors refused to take part in the survey. All together, 1762 women who had delivered during 2009 took part in the survey.

### Data analysis

Included in maternal healthcare services were antenatal consultations, delivery assisted by qualified healthcare personnel and postnatal consultations. The use of healthcare services was defined as being the use and access to healthcare linked to these services at a health facility. ANC was defined as healthcare linked to pregnancy received by a woman at a health facility before labour [4]. Assisted delivery was defined as delivery taking place under supervision of qualified healthcare personnel. Qualified healthcare personnel was considered as all healthcare professionals who have accredited training in healthcare and have the competencies required to enable them to oversee pregnancy, delivery and immediate normal postpartum period, as well as knowing how to identify, treat, and when necessary, take the decision to transfer women or newborn presenting with complications. This category was comprised of doctors and nurses, while traditional midwives were not included [10]. With regard to postnatal consultations, these are planned according to the rule of six: six hours, six days and six weeks after delivery [4]. At least one visit at the facility is expected within a maximum period of 42 days following delivery and at least two visits are expected after this period. Within the framework of this research, we evaluated recourse to the PNC for respective periods of seven days, 28 days (corresponding to the end of the neonatal period) and 42 days [4,11].

Use of healthcare services was defined as use of and access to healthcare linked to these services at a health facility, whereas non-use was defined as the absence of use and the lack of access to healthcare linked to these services. Non-use of ANC, PNC and home delivery were studied as dependent variables. Thus, the women who had not attended ANC were considered as not having used this service; they were compared to those who had attended them at least once during pregnancy. In addition, the women who had delivered at home were compared to those who had delivered at a health facility. All of the women who had not used PNC within seven days, 28 days and 42 days (limit days inclusive) after delivery were compared with those who had used them respectively during the course of these periods.

The different data collected were processed and analysed using Stata 11.0 software. The use of each of the healthcare services was calculated as the proportion of women having used the service in comparison with the total women surveyed. The different quantitative variables (age, gestational age at first visit, frequency of visits, age of the child at the first postnatal visit) were synthesized for their position and dispersion parameters. The comparison of quantitative variables was performed using Student’s *t*-test or the Mann–Whitney test accordingly [12]. The qualitative variables were compared using the chi-square test. The materiality threshold was 5%.

Odds ratios (OR) and their 95% confidence intervals (95%CI) were calculated in order to estimate the strength of association between dependent variables and independent variables.

Logistic regression models were established by progressive selection and only statistically significant variables were conserved. In the tables, the OR (95%CI) derived from final logistic models were presented as well as the p-value of the Wald test. The suitability of the models was verified using the Hosmer and Lemeshow test [12,13]. The materiality threshold was set at 5% for all analyses, which were performed using Stata 11.0 software.

## Results

### Profile of women and households

The average age of the women surveyed was 27 years (SD: 6 years; minimum and maximum: 14 and 49 years); 80% of the women were aged between 20 and 34; almost 10% were under 20 years of age while the same proportion were aged equal to or greater than 35.

Nearly all of the women were married, and only 3.6% were child mothers (63). As regards to the level of education, nearly half of respondents had secondary school education (48.1%), while 24.3% had primary level at most, and 27.6% of the women were of university level.

Overall, 57.9% of the women looked after the household, 27.6% were in sales business, 7.7% worked in the public service and 6.8% carried out work in the fields.

Nearly half of the women were multiparous (48.6%), 30.3% were primiparous and 21.1% were grand multiparous. In the case of multiparous women, 91.9% had a favourable outcome (normal delivery) in the previous pregnancy and 8.1% had had an unfavourable outcome (either a caesarean section, prematurity, still birth, neonatal death or, low birth weight). Twenty-one percent of multiparous women had had complications at the time of previous delivery.

The average size of the households interviewed and the average number of rooms were eight people and three rooms. Overall, 76.8% of the women lived in houses built of durable materials; 72.8% of the households had tap water and 67.3% used electricity for lighting the house. However, coal and wood were the main source of cooking energy for households (86.4%).

The average time between delivery and the survey was 5.5 months (Minimum and maximum: 1–12 months). Statistically, there was no significant difference in this time between healthcare zones, but significant difference was noticed in this median length of time between the Vangu (8.6 ± 3.7 months) healthcare area and all other healthcare areas.

### Recourse to maternal healthcare services

In all, 92.6% of the women surveyed (n = 1762) had attended antenatal care at least once, 93.5% had delivered at a health facility, and 34.6% had attended postnatal consultations in the 42 days following delivery.

### Antenatal care

Table 2 shows that most of the women had attended ANC for reasons essentially linked to the wellbeing of foetus (47.2% for assessment of fetal vitality and 20.2% for determination of the fetal positioning), less than 30% had attended for reasons limited simultaneously to health of the foetus and of the mother (11.8% for vaccination, 7.4% for an insecticide treated net, 5.2% for laboratory examination, and 3.4% for healthcare).

**Table 2:** Reasons for attending versus non-attendance at antenatal consultations in Lubumbashi

**Main reason**	**% (n = 1631)**
ANC (Yes)	
Foetal vitality	47.2
Position of the foetus	20.2
Vaccination	11.8
Laboratory examination	5.2
Treated net	7.4
Healthcare	3.4
No reason	1.3
ANC (No)	% (n = 131)
No reason	44.2
Lack of financial means	26.7
Lack of time	16.0
Distance	4.6
Prohibition by the church or partner	4.6
Accustomed to delivery	3.8

Among the women that did not attend ANC (n = 131), nearly half them reported to have had no reason for attending. The lack of financial means or time, prohibition by the church or partner, as well as the poor perception of women on risks linked to each pregnancy were also reasons given for non- ANC attendance.

In Table 3, it is apparent that, of all of the women who had attended ANC, 98.1%(n = 1580) remembered their gestational age at the start of their ANC visits. Likewise, 94.9%(n = 1548) recalled the frequency of visits made during their last pregnancy.

**Table 3:** Start and frequency of antenatal visits

**Variables**	**Frequency**	**Percentage**
Gestational age at the start of ANC in months (n = 1580)		
<4	808	51.1
≥4	772	48.9
Median (months): Min&Max	4 (1–8)	***
Reasons for the choice of time of visits (n =1580)		
No reason	516	32.7
Malaise	351	22.2
Availability of time	288	18.2
Recommended time	258	16.3
Availability of financial means	167	10.6
Frequency of visits (n = 1548)		
<4	727	47.0
4	451	29.1
≥5	370	23.9
Median: Min &Max	4.0 (1–8)	***
Reasons for more than 5 visits (n = 370)		
No reason	166	44.9
Recommendations	128	34.6
Illness	76	20.5

Min & Max: Minimum and maximum.

The median gestational age at the first ANC visit was four months, but nearly three quarters of these women had no specific motive for attending ANC. However, for 40.4% of the women, it was the onset of malaise during pregnancy and the availability of time which prompted the initial visit; and 16% had done so because they felt it was the recommended time.

As regards to the frequency of visits, 47% of the women had attended ANC less than four times; and the median frequency of visits was four. For the women who had attended ANC five times or more, 44.9% had no specific reason for this, while 34.6% thought it was the recommended frequency, and 20.5% due to malaise felt during pregnancy.

Most of the women (96%) discussed the matter of healthcare education with healthcare personnel at least once during ANC (Table 4). General hygiene, nutrition and the prevention of malaria were However, compared to other matters, prevention of sexually transmitted infections was less discussed (60.9%), the most discussed matters with the women during antenatal consultations.

**Table 4:** Participation in healthcare education by women during the course of antenatal consultations

**Variables**	**Frequency (n = 1631)**	**Percentage (95%CI)**
Healthcare education (all matters)	1566	96.0 (95.1 – 97.0)
Nutrition	1218	74.7
Urogenital infections	1102	67.6
General hygiene	1231	75.5
Sexually transmitted infections/HIV	994	60.9
Malaria	1135	69.6
Childbirth plan (Yes)	1284	78.7 (76.7 – 80.7)
Guidance regarding the place of delivery	803	49.2
Preparations for delivery	1052	64.5
Signs of risk during pregnancy	954	58.5
Signs signalling delivery	1039	63.7
Time of departure to maternity	913	56.0
Examinations (all)	1532	93.9 (92.7 – 95.0)
Height of fundus	1406	86.2
Pelvimetry	1172	71.9
Noise from the heart of the foetus	1438	88.2
Colouration of the conjunctives	1362	83.5
Weighing	1427	87.5
Measuring size	1433	87.9
Taking blood pressure	1408	86.3
Urine	1257	77.1
Examination for lower limb oedema	818	50.2
Blood extraction	1371	84.1

All percentages were calculated according to the number of women who had attended ANC.

Overall, the delivery plan was discussed with most of the women at ANC. Nevertheless, place for delivery was not commonly discussed, and less than 60% of the women were informed about pregnancy complications and their signs, as well as instances when they should go to maternity center or health facility. What were discussed with the majority of the women were preparations for delivery and the signs of labour (Table 4).

With regard to the examinations carried out (physical and laboratory), lower limb oedema was less commonly assessed than other examinations among the women that had used ANC.

Table 5 shows that of all of the services scheduled for ANC, screening for HIV infection and the distribution of treated mosquito nets were the least commonly carried out; 15.5% and 12.8% respectively. For the women that managed to benefit from these interventions, majority got them during the second trimester of pregnancy. For women that had received sulfadoxinepyrimethamine (SP) and tetanus vaccine (TTV), only four out of ten managed to receive two doses.

**Table 5:** Healthcare interventions received during the course of antenatal consultations

**Interventions**	SulfadoxinePyrimethamine %(n = 1631)	Iron % (n = 1631)	Folic acid %(n = 1631)	ITN %(n = 1631)	Mebendazole %(n = 1631)	Tetanus vaccine %(n = 1631)	Test for HIV % (n = 1631)
Coverage	32.7	35.6	29.2	12.8	38.1	60.6	15.5
*95%CI*	*30.4–35.0*	*33.3-38.0*	*27.0 – 31.5*	*11.2 – 14.5*	*35.7-40.5*	*58.2-63.0*	*13.8-17.4*
Start (months)	(n = 533)	(n = 580)	(n = 476)	(n = 209)	(n = 621)	(n = 988)	(n = 253)
1	0.0	2.8	3.2	25.8	1.9	2.2	4.0
2	0.0	6.7	4.2	6.7	6.0	5.3	4.0
3	16.1	11.5	13.4	20.2	11.2	14.4	14.7
4	19.3	17.0	18.1	13.5	16.2	17.1	17.3
≥5	64.6	62.0	61.1	33.7	64.7	61.1	60.0
Frequency (dose)							
1	62.1				82.9	57.2	
2	37.9				17.1	42.8	

ITN: insecticide treated net.

Most of the women who had attended ANC (87.8%) were satisfied with the services; while 3.9% were not satisfied and 8.3% could not pass judgement on the quality of the healthcare received. Of those that reported to have been satisfied, with the ANC services, 46% expressed no reason why they were contented, while 37.3% were fulfilled by existence of friendly healthcare personnel; 9.2% by the healthcare services themselves; and 7.5% by the presence of medicines.

The absence of medicines at ANC (25.4%), the unfriendly relationship of healthcare personnel (12.7%) and lack of cleanliness at health facilities (1.6%) were interpreted by the respondents as signs of poor quality of healthcare, and a reason for non-satisfaction. Of those that were not satisfied by the health care services received (12.2%) majority (60.3%) did not have a reason for their dissatisfaction.

### Assisted deliveries

In total, 1714 women (97.2%) benefited from qualified assistance at the time of delivery. Among the women who benefited from such assistance 3.6% delivered at home. Added to the number of those who did not receive assistance, the proportion of the women who delivered at home was 6.5% in the city of Lubumbashi. The rate of caesarean section among the women surveyed was of 4.5% (75 women).

Among those that delivered at home (6.5%), almost six out of every ten (57.8%) had no reasons for choosing a home birth; while 37.6% had done so due to a lack of financial means, 1.8% due to long distance and lack of transport means to facility. About 0.9% found the healthcare facility staff unfriendly and 1.8% considered it unnecessary to deliver at a healthcare facility as they themselves are more experienced in delivery.

Most home deliveries (56.4%; n = 110) were at the midwife’s home, 21.8% (n = 110) were at the church and 14.6% (n = 110) at the woman’s own home; and 7.3% (n = 110) of the women delivered on their way to maternity.

### Postnatal consultations

In the Table 6, among the women who had not made postnatal consultations, 91% had missed them for no reason. The median time between delivery and the first postnatal visit was 14 days. Up to 42 days after delivery, the median number of visits was three for all of the women; the majority (80.6%) made at most four visits to the services.

**Table 6:** Reasons for not attending and profile of the subjects having attended postnatal consultations

**Postnatal consultations**	**%(n)**
No	65.4(1152)
Main reason	
No reason	91.0
Busyness	3.7
Lack of financial means	3.2
Distance	1.3
Illness	0.8
Yes	34.6(610) †
Time of start of visits (days)	
<7	34.8
8-14	23.6
15-28	9.7
29-42	31.9
(Median, min &max)	14 (1–41)

†: Assessment at 42 days.

### Factors determining the non-use of maternal health services

Table 7 shows that the woman’s age, their marital status, profession, level of education and complications during the previous delivery were not associated with utilization patterns of maternal healthcare services; and the place of delivery was not associated with non-use of PNC. On the contrary, parity was associated with the ANC utilization patterns, where primiparous and grand multiparous women were twice as likely not to attend ANC compared multiparous women. However, more grand multiparous women delivered at a health facility than multiparous women.

**Table 7:** Factors determining non-use of maternal healthcare services: non-adjusted analyses

Variables	n	Antenatal consultations (No versus Yes) n = 1762			Delivery at a health facility (No versus Yes) n = 1762			Postnatal consultations (No versus Yes) n = 1762								
								PNC ≤ 7 days			PNC ≤ 28 days			PNC ≤ 42 days		
		%	OR (95%CI)	p		%OR (95%CI)	p	%	OR (95%CI)	p	%	OR (95%CI)	p	%	OR (95%CI)	p
Age (years)				0.07			0.03			0.21			0.92			0.81
<20	154	12.3	1.9 (1.1-3.3)		4.5	0.6 (0.3-1.4)		89.0	1.1 (0.6-1.3)		75.3	0.9 (0.6-1.4)		66.2	1.0 (0.7-1.5)	
20 - 34	1420	6.8	1.0		6.9	1.0		88.4	1.0		76.6	1.0		65.6	1.0	
≥35	188	8.0	1.2 (0.7-2.1)		2.7	0.4 (0.1-0.9)		84.0	0.7 (0.5-1.1)		76.1	1.0 (0.7-1.4)		63.3	0.9 (0.7-1.2)	
Marital status				0.11			0.97			0.07			0.14			0.29
Married	1699	7.2	1.0		6.2	1.0		87.7	1.0		76.2	1.0		65.2	1.0	
Single	63	12.7	1.9 (0.9-4.0)		6.3	1.0 (0.4-2.9)		95.2	2.8 (0.9-14.1)		84.1	1.7 (0.8-3.5)		71.4	1.3 (0.8-2.3)	
Profession				0.43			0.60			0.15			0.57			
Household	1021	7.0	1.0		6.2	1.0		89.3	1.0		77.3	1.0		65.6	1.0	
Sales	486	9.1	1.3(0.9-2.0)		5.8	0.9(0.6-1.5)		86.6	0.8(0.1-1.1)		76.1	0.9(0.6-1.2)		65.6	1.0(0.8-1.3)	
Agriculture	120	6.7	1.0(0.4-2.0)		5.8	0.9(0.4-2.1)		83.3	0.6(0.1-1.0)		71.7	0.7(0.2-1.1)		60.0	0.8(0.5-1.2)	
Public service	135	5.9	0.8(0.4-1.8)		8.9	1.5(0.8-2.8)		86.7	0.8(0.4-1.3)		75.6	0.9(0.7-1.4)		67.4	1.1(0.7-1.6)	
Level of studies				0.090			0.35			0.77			0.73			0.09
None and primary	847	8.0	1.6(1.0-2.7)		5.8	1.0(0.6-1.7)		88.6	1.1(0.8-1.6)		77.2	1.1(0.8-1.5)		65.3	1.2(0.9-1.5)	
Secondary	429	5.1	1.0		5.6	1.0		87.4	1.0		75.3	1.0		61.8	1.0	
University	486	8.4	1.7(1.0-2.9)		7.6	1.4(0.8-2.4)		87.5	1.0(0.7-1.5)		76.1	1.0(0.8-1.4)		68.7	1.4(1.0-1.9)	
Parity (number)				<0.001			0.04			0.12			0.31			0.34
1	534	9.6	2.0 (1.3-3.0)		5.6	0.7 (0.5-1.1)		89.5	1.4 (0.9-1.9)		78.7	1.2 (0.9-1.6)		67.0	1.1 (0.8-1.3)	
2 - 4	856	5.0	1.0		7.6	1.0		86.3	1.0		75.1	1.0		65.7	1.0	
≥5	372	9.9	2.1 (1.3-3.3)		4.0	0.5 (0.3-0.9)		89.5	1.4 (0.9-2.0)		76.3	1.1 (0.8-1.4)		62.4	0.9 (0.7-1.1)	
Complications at previous delivery				0.16			0.28			0.16			0.85			
Yes	1.057	6.6	1.1(0.7-1.9)		6.2	1.1(0.7-1.9)		88.6	0.7(0.1-1.0)		76.4	0.9(0.8-1.3)		65.9	1.0(0.8-1.3)	
No	286	7.3	1.0		7.0	1.0		84.3	1.0		75.5	1.0		66.1	1.0	
Primigest	419	9.6	1.5(0.9-2.2)		5.7	0.9(0.6-1.5)		89.0	1.0(0.8-1.5)		77.3	1.1(0.7-1.4)		63.7	0.9(0.7-1.2)	
Antenatal consultations			†				0.09			0.006			0.001			<0.001
Yes					6.5	1.0		87.4	1.0		75.5	1.0		64.1	1.0	
No					3.1	0.5 (0.2-1.2)		95.4	3.0 (1.3-8.5)		88.5	2.5 (1.4-4.5)		80.9	2.4 (1.5-3.7)	
Place of delivery			†			†				0.41			0.83			0.98
Healthcare facility	1652							88.1	1.0		76.4	1.0		65.4	1.0	
Home	110							85.5	0.8(0.5-1.4)		77.3	1.1(0.7-1.7)		65.5	1.0 (0.7-1.5)	
Complications at delivery			†			†				0.03			0.020			0.003
No	1687							88.3	1.0		76.9	1.0		66.1	1.0	
Yes	75							80.0	0.5 (0.3-0.9)		65.3	0.6 (0.3-0.9)		49.3	0.5 (0.3-0.8)	
Desire for pregnancy				<0.001			0.93			<0.001			<0.001			<0.001
Partner	83	7.2	0.7 (0.3-1.6)		4.8	0.7 (0.3-2.1)		81.9	0.4 (0.2-0.8)		73.5	0.6 (0.4-1.1)		69.9	0.8 (0.5-1.3)	
Woman	103	2.9	0.3 (0.1-0.9)		6.8	1.0 (0.4-2.5)		83.5	0.5 (0.2-0.8)		70.9	0.5 (0.3-0.9)		54.4	0.4 (0.3-0.6)	
Couple	773	5.3	0.5 (0.3-0.7)		6.1	0.9 (0.6-1.4)		85.1	0.5 (0.4-0.7)		71.9	0.6 (0.5-0.7)		57.6	0.5 (0.4-0.6)	
Accidental	803	10.1	1.0		6.5	1.0		91.9	1.0		81.8	1.0		73.8	1.0	

†: not calculated, same data or no temporal relationship.

The non-use of ANC was not associated with home delivery but was associated with the non-use of PNC. Those women who had not attended ANC during pregnancy were three times more likely not to attend postnatal consultations regardless of the time considered for making the first visit. Likewise, the desire for pregnancy (planned pregnancy) was associated with the use of ANC or PNC and the women who had had an unplanned or unwanted conception were more likely not to attend ANC or PNC in comparison with those who had planned their pregnancy alone or in a couple.

Table 8 highlights the fact that only parity and the desire for pregnancy were associated with the ANC utilization patterns. However, parity was not associated with home delivery or use of PNC. The factors associated with non-use of PNC in univariable analysis were also associated in multivariable analysis.

**Table 8:** Factors determining non-use of maternal healthcare services: OR adjusted by logistic regression

	Antenatal consultations (No versus Yes) n = 1762			Delivery at a health facility (No versus Yes) n = 1762			Postnatal consultations (No versus Yes) n = 1762								
							PNC ≤ 7 days			PNC ≤ 28 days			PNC ≤ 42 days		
	aOR	95%CI	p	aOR	95%CI	p	aOR	95%CI	p	aOR	95%CI	p	aOR	95%CI	p
Age (year)			0.07			0.05	‡			‡					0.81
<20	1.5	0.9-2.8		0.6	0.3-1.5								0.9	0.6-1.3	
≥35 vs 20-34	0.9	0.5-1.8		0.4	0.2-1.1								0.9	0.7-1.3	
Single vs Married	1.1	0.5-2.5	0.14	1.0	0.4-3.1	0.97	‡			‡			‡		
Profession			0.45	‡			‡			‡			‡		
Sales vs Household	1.3	0.9-2.0													
Agriculture vs Household	1.1	0.5-2.3													
Public service vs Household	0.8	0.4-2.8													
Level of studies			0.09			0.35	‡			‡			‡		
None and primary vs Secondary	1.4	0.8-2.3		1.1	0.7-1.9										
University vs Secondary	1.4	0.8-2.5		1.5	0.9-2.6										
Parity (number)			<0.001			0.05	‡			‡			‡		
1 vs 2-4	1.8	1.1-2.8		0.7	0.3-1.6										
≥5 vs 2-4	2.0	1.2-3.4		0.6	0.3-1.1										
Complications at previous delivery	‡					0.79	‡			‡			‡		
Yes vs No				1.1	0.7-1.9										
Primigestvs No				1.2	0.5-2.8										
Without ANC vs ANC	†			0.5	0.2-1.3	0.09	2.6	1.1-6.1	0.002	2.3	1.3-3.9	<0.001	2.1	1.3-3.3	<0.001
Home delivery (Yes vs No)													1.0	0.7-1.5	0.98
Complications at current delivery (Yes vs No)	†			†			0.6	0.3-0.9	0.04	0.6	0.4-0.9	0.03	0.5	0.3-0.8	0.004
Desire for pregnancy			<0.001			0.93			<0.001			<0.001			<0.001
Partner vs Unwanted	0.7	0.3-1.8		0.7	0.3-2.1		0.4	0.2-0.8		0.6	0.4-1.1		0.9	0.5-1.4	
Woman vs Unwanted	0.2	0.1-0.8		1.0	0.4-2.3		0.5	0.3-0.8		0.6	0.4-0.9		0.4	0.3-0.7	
Couple vs Unwanted	0.5	0.3-0.8		0.9	0.6-1.4		0.5	0.4-0.7		0.6	0.5-0.7		0.5	0.4-0.6	

‡: not included in the model; †: not calculated, same date or no temporal relationship; vs: versus (comparison group follows “versus”).

Indeed, considered in comparison with women who had two to three deliveries, primiparous and grand multiparous women were twice as likely not to use ANC during their pregnancy. This association is also notable in the use of PNC. The women who had an accidental pregnancy were more likely not to use ANC or PNC than those who had planned their pregnancy alone or in their couple.

Besides, the women who had not attended ANC were also more likely not to attend PNC. The process of delivery was also associated with the use of PNC. Those women who had experienced some complication during delivery frequented PNC more than those who had had normal deliveries.

## Discussion

In Lubumbashi, the majority of pregnant women go for antenatal consultations and give birth in healthcare facilities, but still make very little use of postnatal consultations.

In fact, though women do go for antenatal consultations, most of those who used this service were not properly aware of its advantages. The women considered that such services had more advantages for the foetus than for themselves (Table 2). This lack of knowledge among women with respect to the twofold advantages of this service (maternal, as well as foetal and neonatal), reflects a deficiency in information due to an absence of continuum in these services between healthcare facilities and the community [14-19]. These findings corroborate well with the observations of Anya et al. [20] who noted in Gambia that the source of information on antenatal consultations was mainly women, close acquaintances (mother-in-law and friends), and in a very small proportion, health personnel. From the above, it follows that the extent of utilising NPC and health treatment offered during these consultations, is an indication of the information available to women [18-21]. The better informed women are, the more demanding they will be regarding the nature and quality of the healthcare that they need. They will thus make favourable decisions in terms of the start and frequency of their antenatal visits.

Concerning the start of ANC, it was noted that most women started late with these visits (in the second trimester of pregnancy), and that less than half completed the recommended number of visits throughout their pregnancy [11,22]. Moreover, it also appeared from our findings that the women did not follow a specific schedule for their ANCs. Most of them started their antenatal visits only when they had the financial means or time, or felt unwell (Table 3); only 20% of the women started with antenatal visits according to a planned schedule. This behaviour, which was also observed in several other African regions [4], can be attributed to the lack of adequate information on the content and schedule of antenatal visits [21], and constitutes a serious barrier to the utilisation of all the interventions recommended for efficient pregnancy care.

It also appears from the study that HIV and planning for delivery were not frequently discussed between health personnel and pregnant women during antenatal visits. The proportions of women who had access to these services were low, and were similar to those observed in various other African countries where health systems appear unstable. In such context, health personnel are inclined to focus on more lucrative activities than communicating with pregnant women [23]. Therefore, the small amount of communication between health personnel and pregnant women regarding their delivery plan can be explained by the low percentage of women who complete their series of antenatal visits, given that this issue is normally discussed on the last visit [24-26]. The scarcity of information that could raise awareness of HIV is in turn due to a very low coverage of PMTCT activities in healthcare facilities (less than 15% in Katanga) [4,5].

Besides the deficient raising of awareness and delivery planning, women did not undergo all of the recommended physical examinations and laboratory tests during antenatal visits. The proportion of those who underwent examinations for oedema in the lower members was low compared to other towns in Africa [4,16]. This attitude, which is a barrier to detecting potential risk factors, deprives women of the chance to benefit from adequate healthcare when needed.

The proportions of women who had used sulfadoxine-pyrmethamine (SP), iron, folic acid, or insecticide-treated mosquito nets (ITN), and those who were vaccinated against tetanus or had been tested for HIV, were low and of a great cause for concern, since the antenatal visits had not presented an opportunity to provide them with appropriate healthcare. These were, on the contrary, missed opportunities during which the women had limited contact with the health facility and personnel, without ever benefiting from health interventions that nevertheless were proven to be effective in preventing and treating most of the problems (such as anaemia, malaria, HIV infection and tetanus) encountered in pregnant women in poor, tropical areas such as the DRC [4]. The main reasons for these missed opportunities are on one hand, the lack of availability of such treatments, and on the other hand, the women’s lack of financial means.

Certain health treatments such as SP or tetanus vaccination should be given to women free and administered under observation. In the case of iron, folic acid and ITN, which they must continue to use at home, healthcare services must be organised in such a way that they can be provided to women free, or at affordable prices so that they do not need to make the extra effort of procuring them elsewhere. However, from lack of such inputs in healthcare facilities, health personnel are often obliged to issue prescriptions to pregnant women, without knowing whether they will follow them. Very few women follow such prescriptions if especially they consider that their state of health does not require them to take the medicines, or they do not have the means to buy them. In India, for instance, Lim et al. noted an increase in the use of health treatments in areas where they were freely available, as opposed to areas where pregnant women or their families had to pay for them [23]. In Lubumbashi, subjecting pregnant women to additional costs (besides that of the consultation sheet) before they access to tetanus vaccination for instance, contributed enormously to limiting their access to such treatments.

We also observed in the course of this study that most women gave birth at healthcare facilities. Regarding home births, women mentioned lack of financial means as the main reason for not going to a healthcare facility, but a considerable proportion of the women considered it less important to give birth in a healthcare facility, given the experience they had already gained from previous deliveries. This behaviour, which is not beneficial to women, goes hand in hand with a misconception of the risks of childbirth, and can be attributed to insufficient or poor communication between healthcare facilities and the community in general, and women in particular [24]. Mobilising the community and creating interaction between healthcare facilities and the community are indispensable and a matter of urgency, in order to reinforce the information that is given to women, and improve their perception of the risks involved in pregnancy and childbirth.

In contrast to the observations made by Chenge et al. [7], the Caesarean-section rate in our study was 4.5%. This rate is nearly three times higher than the one reported by the aforementioned author, but twice lower than in other African countries [4]. The difference in the two rates that were calculated in the same city is due to the calculation methods. Chenge et al. estimated this rate according to the number of expected births in Lubumbashi, whereas the rate in the present study was calculated directly among women who participated in the study. However, seen as an indicator for assessing the level of maternal mortality, the Caesarean-section rate remains low and indicates an under-utilisation of this procedure in Lubumbashi [27].

Postnatal visits in turn are rarely used by women in Lubumbashi, even when one allows for a 42-day lapse after birth [4]. Postnatal visits were more frequent at 42 days than at seven days, since the 42^nd^ day coincides with the start of preschool visits [28]. Superstition to the fact that newborns will be exposed to demonic influences and witchcraft if taken outside the house in the first month has been mentioned in other areas as a reason for delaying the first visit. Visits starting at 42 days therefore correspond to the moment when mothers consider that their child is no longer in such danger [28]. These beliefs, combined with a lack of motivation to go for postnatal visits, also indicate deficiency in the information given to women.

Regarding factors that determine the utilisation of maternal healthcare services, we observed that primiparity, grand multiparity, and unwanted pregnancies issues associated with the non-utilisation of antenatal visits. Reasons suggested for the non-utilisation of postnatal visits were the lack of antenatal visits, the absence of complications during birth, and unwanted pregnancies.

In fact, we noticed that women who were pregnant for the first time, and those who had been pregnant many times, were at greater risk of not using ANC than those who had been pregnant a few times. These observations correspond with findings from other studies [29-43]. The tendency for first-time pregnant women to make less use of ANC could be explained, on the one hand, by the lack of information on pregnancy management (e.g. presence of maternity facilities that are integrated in the community and destined for women of childbearing age). On the other hand, women who had been pregnant many times were less inclined to go for antenatal visits due to their misconception of the risks of pregnancy, because of experience gained from previous pregnancies and births [44].

The utilisation of antenatal consultation did not affect the fact of giving birth at a healthcare facility or not. However, it did influence the likelihood of going for postnatal visits. This influence is due to the climate of trust generated by the friendly relationships that are gradually built up between women and health staff, and which becomes a motivational factor for the women to go for postnatal visits [3]. Complications arising during the delivery have an effect on the utilisation of postnatal consultations, due to the women’s perception that such complications present a threat to maternal and neonatal survival. In this context, postnatal visits become a necessity in order to improve the prognosis. This observation suggests therefore that the message given by health personnel encouraging women to make use of postnatal consultations is selective. Health personnel tend to insist more on postnatal visits for women who had complications during the delivery, than for those who gave birth without any problems.

Birth planning was also associated with the utilisation of mother-child healthcare. In the present study, women who planned their pregnancy, whether alone or with their partners,were more inclined to make use of pre- and postnatal consultations than those whose pregnancy was unplanned. This association shows the need to have and reinforce family-planning services. From a psychological viewpoint, a planned pregnancy is usually better accepted by the women, which motivates her to adopt a favourablebehaviour pattern, for instance making use of healthcare services at recommended times or when there is a problem, in order to benefit from treatment that is appropriate for the situation [4,33].

This study, which was carried out in an essentially urban environment, has certain limitations that must be pointed out.

Firstly, we did not investigate the role of insufficient financial means, which is generally recognised as a barrier to the utilisation of healthcare services [29]. The fact that the impact of this factor on the utilisation of mother-child healthcare is not mentioned, does not mean that it is not a determining factor for these services. In fact, women frequently mentioned it as being an obstacle to going for antenatal visits as well as giving birth at a health facility. The absence of this element among the determining factors is due to a high rate of respondents who did not answer questions that could have been used for calculating the poverty index of households. This attitude was explained by the fear of being burgled or threatened, according to the women.

Secondly, we considered that distance, which was not included in the determining factors for this study, was not an obstacle to the utilisation of services, given that the city of Lubumbashi is divided into healthcare zones that are mainly urban, where almost all health areas are operational and easily accessible. Nevertheless, this appreciation does not exclude considerations related to preference. A woman may prefer a healthcare facility that is further from her home, rather than one that is closer by, due to factors like cleanliness, presence of medicines, or the presence of friendly staff.

Thirdly, aspects such as community participation and balanced social relationships between men and women could also influence the use of mother-child healthcare services.

In Lubumbashi as in the rest of the DRC, activities relating to healthcare services for mothers, newborns and infants are generally delivered as clinical or outreach services; there are no family- or community-based services; nor are there any cooperation mechanisms between the healthcare system and the community. In this context, the community plays a passive role in the service offer, which is limited to paying fees for services received. The fact of not associating with the healthcare service offered could account for the lack of information observed in this study, and explain why the quality of these services does not improve, when the community has no mechanism for holding the healthcare system to account [7].

Moreover, social imbalance in favour of men could also explain the rate of healthcare use. In Lubumbashi for instance, only one woman for every three men has a paid job [5]. This ratio means that most women are financially dependent on men. The lack of autonomy could also be an element that determines the use of mother and child healthcare services, since the decision to make use of such a service is often left to the man. Therefore, if the man does not participate in mother-child healthcare activities, it is natural that his ignorance, combined with the decision-making power of which he holds the monopoly in the household, could have a negative impact on the use of services.

These considerations imply that information to women may well be improved, but that if the participation of the community and the autonomy of women are not reinforced, then neither could the use and quality of mother-child healthcare services be improved.

Such biases could for instance have influenced the gestational age at the first antenatal visit, for in Lubumbashi, this age is mainly determined according to the date of the last menstrual period, which some women may have forgotten. Moreover, since women’s statements were confirmed by the information recorded in ANC records and files, we do not exclude the possibility that the recording of certain data may have been forgotten by the staff of this service, even if in most cases, the information given by the women correlated with that found in the documents.

Fourthly, we do not exclude the possibility that these findings may be limited by memory bias which is inherent to surveys based on questionnaires, given the fairly large number of months between delivery and the date on which the women responded to the questions.

## Conclusions

In Lubumbashi, a significant proportion of women continue not to make use of healthcare services during pregnancy, as well as during and after childbirth. Women giving birth for the first time, those who have already given birth many times, and women with an unwanted pregnancy, made less use of ANC. Moreover, women who had not gone for ANC rarely came back for postnatal consultations, even if they had given birth at a healthcare facility. Similarly, those who gave birth without complications, less frequently made use of postnatal consultations. As with ANCs, women with unwanted pregnancies rarely went for postnatal visits.

In addition to measures aimed at reinforcing women’s autonomy, efforts are also needed to reinforce and improve the information given to women of childbearing age, as well as communication between the healthcare system and the community, and participation from the community, since this will contribute to raising awareness of safe motherhood and the use of such services, including family planning.

## Competing interests

The authors declare that they have no conflict of interest in the present research.

## Authors` contributions

ANML and FMK carried out the study and participated in the statistical analysis and procedures. MWD, PD coordinated and participated in the design of the study, statistical analysis and the drafting of the manuscript. All the authors read and approved the final version.

## Pre-publication history

The pre-publication history for this paper can be accessed here:

http://www.biomedcentral.com/1471-2393/12/66/prepub
